# Transfer of working memory training to the inhibitory control of auditory distraction

**DOI:** 10.1007/s00426-020-01468-0

**Published:** 2021-01-15

**Authors:** Florian Kattner

**Affiliations:** Institute for Psychology, Technical University of Darmstadt, Alexanderstr. 10, 64283 Darmstadt, Germany

**Keywords:** Working memory training, n-back task, Inhibitory control, Distractor interference, Irrelevant speech effect

## Abstract

Extended working memory training with the dual *n*-back task has been shown to improve performance on various untrained cognitive tasks, but previous findings were inconsistent with regard to the extent of such transfer. The dual *n*-back training task addresses multiple components of working memory as sequential information from two different stimulus modalities needs to be simultaneously encoded, maintained, continuously monitored and updated in working memory while irrelevant information needs to be inhibited. However, it is unclear which executive functions account for the observed transfer effects. In this study, the degree of inhibitory control required during training was manipulated by comparing two versions of the dual *n*-back task in which participants are asked to either respond or withhold a response on the less frequent trials when an item was identical to an item *n* trials back. Eight 80-min sessions of training with adaptive versions of both *n*-back tasks were shown to improve working memory updating. Moreover, in contrast to the standard *n*-back task, training on the inhibitory *n*-back task was found to reduce the interference in working memory produced by task-irrelevant speech. This result suggests that enhanced demand for inhibitory control during training enables transfer to the inhibition of distractor interference, whereas the standard *n*-back task primarily affects working memory updating. The training effects did not transfer to the inhibition of spatially incompatible responses in a Simon task, and it yielded no far transfer effects to untrained executive functions or measures of fluid intelligence.

## Transfer of working memory training to the inhibitory control of auditory distraction

Working memory refers to a cognitive system of limited capacity which enables temporal storage and processing (e.g., manipulation, monitoring) of information to support thought and action processes (see Baddeley 2003; Cowan 2017; Miyake and Shah 1999). It has been shown that individual differences in the capacity of working memory are related to a number of complex cognitive or verbal abilities, such as reasoning (Fry and Hale 1996; Kyllonen and Christal 1990), problem-solving and general intelligence (e.g., Conway et al. 2003; but see Harrison et al. 2013), reading comprehension (Daneman and Carpenter 1980; Engle et al. 1991), and selective listening in cocktail-party situations (Conway et al. 2001). Working memory impairment, on the other hand, has been associated with attention deficits and learning disabilities (Alloway 2009; Martinussen et al. 2005). More recently, several studies demonstrated that working memory capacity can be enhanced through extensive cognitive training, both in children and adults, leading to improvement on various cognitive tasks addressing reading comprehension, executive control, episodic memory, or fluid intelligence (Buschkuehl et al. 2008; Chein and Morrison 2010; Dahlin et al. 2008a, b; Jaeggi et al. 2008, 2010; Klingberg et al. 2002; Salminen et al. 2012; Schmiedek et al. 2010; Thorell et al. 2009). Several well-controlled studies, however, failed to replicate these widespread transfer effects resulting from working memory training (Melby-Lervåg and Hulme 2013; Redick et al. 2013; Thompson et al. 2013). Therefore, reviews and meta-analyses on the efficacy of working memory training drew rather inconsistent conclusions (Au et al. 2015; Dougherty et al. 2016; Karbach and Verhaeghen, 2014; Melby-Lervåg et al. 2016; Melby-Lervåg and Hulme 2013; Soveri et al. 2017; von Bastian and Oberauer 2013b). There is still an ongoing debate regarding the specific cognitive functions that benefit from working memory training, and to what extent the training-related improvement of these functions yields transfer to untrained tasks that require more generalized cognitive abilities, such as cognitive flexibility, problem-solving, or fluid intelligence. From the empirical data available, it can be concluded that transfer of working memory training is more likely in transfer tasks that are structurally similar to the trained tasks (near transfer) than when the transfer tasks share only a few features with the trained task (far transfer), but there is still very little understanding of the exact cognitive mechanisms and components of working memory that enable transfer (Gathercole et al. 2019; Shipstead et al. 2010; Simons et al. 2016).

Most models of working memory distinguish (a) one or multiple storage buffers or maintenance components from (b) a component for executive control which enables monitoring and manipulation of the stored information (Baddeley 1996, 2003; Baddeley and Hitch 1974; Engle 2002; Miyake and Shah 1999; Oberauer et al. 2000). Cognitive training tasks, such as the dual *n*-back task, which has been shown to successfully enhance working memory capacity (i.e., the number *n* of items to-be-updated in working memory; see Jaeggi et al. 2008), typically require both maintenance and executive control (e.g., updating) of the information in working memory, but it is still unclear which executive functions benefit most from cognitive training, and how the training-related improvement is related to transfer. Working memory updating and monitoring, set shifting (i.e., cognitive flexibility or task-switching), and inhibition were found to be the three major functions of executive control, which are involved in many cognitively demanding tasks (Miyake et al. 2000), but a majority of studies on working memory training seem to have used tasks that primarily require the updating and monitoring component (e.g., Dahlin et al. 2008a, b; Jaeggi et al. 2008; Kühn et al. 2013; Lilienthal et al. 2013; Salminen et al. 2016). In a typical dual *n*-back task, participants are presented with two running sequences of stimuli (auditory and visual) from which the items of the last few (*n*) trials only need to be memorized. The participants’ task is to indicate whether any of the two items on the current trial is identical to one of the items that were presented exactly *n* trials before. Hence, it is required to continuously monitor and update the information to be maintained in working memory, but the task may also involve inhibition of currently irrelevant items and attention shifting between the two sequences or stimulus modalities. More specifically, it has been suggested that the *n*-back task requires not only encoding, storage, and rehearsal of items, but also discarding (inhibition) of previously encoded items and repositioning (updating) of the to-be-remembered information in working memory (Postle et al. 2001). While the empirical results are still scarce and also inconsistent, there is some evidence suggesting that extended training on the dual *n*-back task does indeed improve updating and monitoring, whereas it may not necessarily generalize to other functions of executive control, such as set shifting or inhibition (Dahlin et al. 2008a, b; Salminen et al. 2012; von Bastian and Oberauer 2013a).

The purpose of the present study is to investigate whether working memory training can be used to enhance the inhibitory control function of working memory. Individual differences in the strength of inhibitory control were shown to predict both the development and the age-related decline of cognitive abilities (Diamond and Gilbert 1989; Hasher and Zacks 1988; Salthouse and Meinz 1995). These findings suggest that inhibitory control may benefit also from a cognitive training, which might have important implications in particular for maintenance of inhibition in the older age. However, it has been argued that inhibition may not be a unitary mechanism, but to refer to three functionally distinct processes (see Friedman and Miyake 2004): (1) suppression of pre-potent or automatic responses (as in a Stroop task; Stroop 1935), (2) inhibitory control of the interference produced by irrelevant stimuli (as in a flanker task; Eriksen and Eriksen 1974; or in an “irrelevant sound paradigm”; Jones and Macken 1993; Salamé and Baddeley 1982), and (3) inhibition of information in memory (e.g., to avoid proactive interference). It has been found that inhibition of pre-potent responses and inhibition of irrelevant stimuli (interference control) may be closely related, whereas inhibition of proactive interference seems to be a separate process (Friedman and Miyake 2004).

While there is some indication that pre-potent response inhibition can be improved with practice (in particular when combined with transcranial direct current stimulation; Ditye et al. 2012), very little is known about the possible effects of an extended working memory training on the other forms of inhibitory control. Here, the effect of two different types of working memory training, varying in the degree of inhibitory control required, were compared with regard to their transfer effects on (a) the ability to suppress pre-potent responses (response inhibition) and (b) the ability to inhibit interference from irrelevant auditory information (resistance to auditory distraction). Specifically, one group of participants was trained on a standard dual *n*-back task which is supposed to involve primarily updating and monitoring of contents in working memory (Braver et al. 1997; Jaeggi et al. 2007), and possibly to some extent other inhibitory control processes, such as the inhibition of irrelevant stimulus information (Postle et al. 2001). To experimentally enhance the degree of inhibitory control involved in the dual *n*-back, a second group was trained on an “inhibitory” version of the dual *n*-back task (inhibitory *n*-back) in which responses had to be given predominantly, and participants had to occasionally inhibit the response depending on the current information held in working memory (i.e., on “*n*-back trials”). Both types of *n*-back training are expected to enhance working memory updating skills which were tested with an untrained visual updating task before and after training (adopted from Dahlin et al. 2008a). Moreover, assuming that response inhibition and resistance to distractor interference are closely related (Friedman and Miyake 2004), any training-related improvement on the inhibitory dual *n*-back task may be expected to induce more transfer to performance in other tasks that require either the suppression of pre-potent responses or inhibitory control of irrelevant stimuli, compared to the standard dual *n*-back task with lower demands for inhibition. Therefore, transfer of the two types of working memory training was assessed in terms of both response inhibition and the degree of interference produced by auditory distractors. In addition, far transfer was tested for unrelated executive functions (i.e., task-switching) and more generalized cognitive abilities (i.e., problem-solving skills related to fluid intelligence) for which transfer was reported previously (e.g., Jaeggi et al. 2008).

Generalization to response inhibition was assessed with the Simon task (Hedge and Marsh 1975) in which a target is presented at a location that is either spatially compatible or incompatible with the location of the response. Specifically, on compatible trials a response needs to be made by the hand that corresponds to the location of the target (the pre-potent response), whereas on incompatible trials, the response needs to be made by the other hand and the pre-potent response needs to be inhibited. Typically, response time increments are observed on incompatible trials, as compared to compatible trials (the Simon effect). The inhibitory working memory training could be expected to affect response inhibition: If the training-related enhancement of inhibitory control enhanced the ability to suppress pre-potent, dominant, or automatic responses (Friedman and Miyake 2004), then reduced Simon effects should be observed at post-test in the inhibitory *n*-back group.

In addition, transfer of the inhibitory training could be expected also with regard to inhibitory control of auditory distraction. It is well known that task-irrelevant sound, such as speech or random tone sequences, disrupts performance in serial short-term memory tasks (e.g., Colle and Welsh 1976; Jones et al. 2004; Jones and Macken 1993; LeCompte et al. 1997; Salamé and Baddeley 1982). While these disruptions were originally explained with speech-related interference-by-content in the “phonological loop” (Baddeley and Hitch 1974; Salamé and Baddeley 1982), it has been shown later that similar disruption can be produced also by non-phonological sound (e.g., changing tones; Jones and Macken 1993), and it has been suggested that the interference may be specific to the processing of serial order in short-term memory (e.g., Jones and Macken 1993, 1995). More specifically, according to the object-oriented episodic record account (Jones et al. 1996), auditory distraction is assumed to be a by-product of perceptual organization processes which enable the segregation and grouping of auditory objects (during auditory scene analysis; Bregman 1990). Any change in the state of background sound is expected to give rise to the formation of a new auditory object, which is automatically linked to the previous objects (using “pointers”), thus creating an ordered stream. In a serial recall task, articulatory rehearsal can be used (as a motor planning process) to deliberately form and refresh links between to-be-remembered items, thus enabling the maintenance and retrieval of serial information. However, automatic processes of auditory perceptual organization form additional links between task-irrelevant changing-state sounds, which then interfere with the deliberate motor planning and rehearsal processes during serial recall. In line with this interference-by-process account (Hughes and Marsh 2017; Jones et al. 2004; Jones and Macken 2018), it has been found that the degree of distraction increases with the magnitude (e.g., the distance in pitch between successive tones; Jones et al. 1999) and the number of changes between successive task-irrelevant auditory events within a given time interval (i.e., the word/token dose effect; Bridges and Jones 1996; Tremblay and Jones 1998, Exp. 5). In addition, changing-state sound (speech or varying tones) was found to disrupt performance in a serial recall task, but not in tasks that do not require serial-order processing (e.g., the “missing-item task”; Beaman and Jones 1997; Hughes et al. 2007; Jones and Macken 1993), unless participants happen to adopt a serial rehearsal strategy (Beaman and Jones 1998; Hughes and Marsh 2020b). In addition to this task-specific interference with serial-order processing, it has been proposed more recently that auditory distraction may arise also from attentional capture, with meaningful or acoustically deviating sounds diverting attention from the focal task (see the “duplex-mechanism account”; Hughes 2014; Hughes et al. 2005). In contrast to interference-by-process, this form of distraction appears to be less specific to serial-order processing (affecting performance also in non-serial short-term memory tasks; e.g., the “missing-item task”, Hughes et al. 2007; Vachon et al. 2017), and it may be more susceptible to cognitive control than interference-by-process (Hughes et al. 2013; Hughes and Marsh 2020a). Moreover, it has been reported that the degree of attentional capture elicited by auditory deviants, but not the changing-state effect (indicating interference-by-process), was related the participants’ working memory capacity (Hughes et al. 2013; Sörqvist et al. 2010)(but see Körner et al. 2017). It is not entirely clear to what extent the disruptive effect of irrelevant speech on serial recall is caused by acoustical interference with serial-order processing and attentional capture, but there is evidence suggesting that at least meaningful speech (e.g., full sentences as compared to lists of changing-state syllables or words) may disrupt performance through both mechanisms (see Bell et al. 2017; Hughes and Marsh 2020b). Moreover, the findings of reduced disruption of serial recall (1) after repeated presentation of the same stream of irrelevant speech (i.e., habituation; Banbury and Berry 1997; Bell et al. 2012), (2) in blind listeners with enhanced auditory processing abilities (Kattner and Ellermeier 2014), and (3) following a specific training of auditory attention (Kattner and Ellermeier 2020) suggest that disruptive effect of irrelevant speech can be partially attributed to the diversion of attention.

In the present study, the transfer of cognitive training was assessed in terms of the disruptive effect of task-irrelevant speech, compared to noise, on serial recall. If the disruptive effect of speech depended on general working memory capacity, then it would be expected that both cognitive trainings with the dual *n*-back task will reduce distraction. In contrast, if auditory distraction was specifically related to inhibitory control of irrelevant sound, then the inhibitory *n*-back training should result in greater attenuation of auditory distraction than the standard *n*-back training with less demands for inhibitory control. Specifically, the inhibitory *n*-back training might enhance the ability to resist or resolve interference from the external environment (Friedman and Miyake 2004). In line with the duplex-mechanism account of auditory distraction, it could be argued that attentional capture by irrelevant speech is likely to depend on inhibitory control, whereas the disruption due to changing-state sound (in irrelevant speech) should not depend on any form of cognitive control (Hughes 2014; Hughes et al. 2013). Therefore, enhanced inhibitory control (or resistance to interference from the external environment) could be expected to prevent the diversion of attention by irrelevant speech, whereas the presumably uncontrollable disruption due to the changing-state nature of speech should remain. A training of inhibitory control should thus lead to an attenuation, but not to a full elimination of the irrelevant speech effect. Alternatively, it could be argued also that enhanced inhibitory control of irrelevant changing-state sound (e.g., inhibiting the formation of irrelevant auditory streams) may reduce the specific interference between auditory grouping and the seriation process, which might then lead to a stronger attenuation or even an elimination of the irrelevant speech effect.

In addition to the transfer effects on performance in tasks that involve similar executive functions as the training tasks—working memory updating, suppression of pre-potent responses (Simon effect), and resistance to interference by irrelevant speech—the present study also tested the possibility of far transfer effects on (a) the response-time costs resulting from task-switching (indicating cognitive set-shifting abilities; Rogers and Monsell 1995) and (b) general problem-solving capabilities, which are related to fluid intelligence (Jaeggi et al. 2008).

## Methods

### Participants

Seventy-four participants (50 women and 24 men) were recruited at the campus of the Technical University of Darmstadt. Four additional participants did not complete the training sessions (and the post-test) and their data could not be included in the analyses. Ages raged between 18 and 62 years (*M* = 24.7; *SD* = 7.5). Participants were randomly assigned to either the standard *n*-back training group (*n* = 23; 17 women; 19 – 38 years, *M* = 23.4; *SD* = 4.3), the inhibitory *n*-back training group (*n* = 25; 16 women; 18 – 62 years; *M* = 25.0; *SD* = 10.0), or the no-training passive control group (*n* = 26; 17 women; 18 – 54 years; *M* = 25.5; *SD* = 7.0). There were no significant age differences between groups, *F*(2,71) = 0.48; *p* = 0.62. All participants reported normal hearing and normal or corrected-to-normal vision. Student participants were compensated with course credits.

A sensitivity analysis revealed that the sample size of *N* = 74 is sufficient to demonstrate an interaction in the present 3 (group) × 2 (pre/post) design with a statistical power of 95% assuming an effect size of *f* = 0.23 or larger (*α* = 0.05). Therefore, the present sample size should provide sufficient power to detect the previously reported transfer effects of a dual *n*-back training on working memory updating (*f* = 1) (*η*^2^_p_ = 0.50 for the interaction using a similar 3 × 2 design; Salminen et al. 2016; p. 10,202) as well as the possible far transfer effect on fluid intelligence *f* = 0.27 (*η*^2^_p_ = 0.07 for the interaction between group and test-session; Jaeggi et al. 2008; p. 6830).

### Apparatus

The experiment was conducted in a single-walled sound attenuated listening booth (Industrial Acoustics Company, Niederkrüchten, Germany). Visual stimuli were presented on a 17-inch LCD monitor, and participants were seated at approximately 59 cm viewing distance. Sounds were D/A converted at 44.1 kHz (16 bits) by a RME multiface II sound card (Audio Ag, Haimhausen, Germany) and passed through a Behringer HA 800 Powerplay PRO-8 headphone amplifier (Behringer, Zhongshan, China) before being played diotically via Beyerdynamics DT-990 headphones (Beyerdynamic GmbH, Heilbronn, Germany). The experimental routines were programmed in MATLAB (Mathworks, Natick, MA) utilizing the Psychophysics toolbox 3.0 extensions (Brainard 1997; Kleiner et al. 2007; Pelli 1997).

### Procedure

For all three groups, the experiment consisted of a pre- and post-test session. The two training groups (*n*-back and inhibitory *n*-back) completed eight 90-min training sessions between the pre- and post-tests, whereas there were no training sessions for the passive control group. The time between pre- and post-tests has intended to be about two weeks, but individual time constraints and preferences resulted in an average interval of 16.6 days (*SD* = 4.1) in the *n*-back group, 17.2 days (*SD* = 6.0) in the inhibitory *n*-back group, and 10.7 days (*SD* = 3.8) in the control group. In both training groups, two adjacent training sessions were separated by at least one and no more than two successive days without training.

### Pre–post tests

During the pre- and post-test sessions, participants completed five different tasks in counterbalanced order (using a Latin square design). It took participants about 60 min to read the instructions and complete (a) 25 runs of working memory updating (approx. 10 min), (b) 24 trials of verbal serial recall (approx. 10–15 min), (c) 200 trials of the Simon task (approx. 7 min), (d) 200 trials of task-switching (approx. 11 min), and (e) 18 problems from the Raven’s advanced progressive matrices (max. 10 min).

In the *working memory updating task* (Dahlin et al. 2008a), there were 25 runs in which a variable number of colored circles were presented successively in the center of a black screen (1250 ms per circle, each preceded by a 1000 ms blank screen). For each run, the colors were randomly drawn from nine possible colors (red, green, blue, yellow, pink, cyan, purple, orange, and grey). After a randomly drawn number of trials (between 5 and 12), participants were asked to recall the colors of the last four circles of the series in correct serial order by clicking on the respective colors in a 3 × 3 response matrix showing the nine colors (red, green and blue in the top row, yellow, pink, and cyan in the middle row, and purple, orange, and grey in the bottom row). Clicked responses could not be corrected, and feedback indicating the number of correctly recalled colors was presented on the screen immediately after the fourth response for 1500 ms before the next run started.

In the visual *Simon task* (Hedge and Marsh 1975), each trial started with a central fixation cross for 500 ms, followed by either a square or a circle (diameter of 3° visual angle) in blue or green color which was presented either 5° to the left or 5° to the right of fixation. Participants were instructed to respond to the color of the stimulus by pressing the ‘A’ key with the left index finger for blue stimuli (left side of the keyboard) and the ‘L’ key with the right index finger for green stimuli (right side of the keyboard), ignoring both the shape and the position of the stimulus. The stimulus-response mappings remained constant across the entire task. In half of the trials, the position of the stimulus was spatially compatible with the response (i.e., a blue stimulus on the left or a green stimulus on the right), and in half of the trials, the position was spatially incompatible (i.e., a blue stimulus on the right or a green stimulus on the left). Participants were instructed to respond as fast as possible while avoiding errors. Both the accuracy and the response time (in ms) were shown as feedback after each response that was given within 750 ms (e.g. “Richtig! 326 ms” [Correct! 326 ms]). For response times longer than 750 ms, participants were only prompted to be faster (“Zu langsam!” [Too slow]). In either case, feedback was presented for 750 ms before the next trial started.

In the *verbal serial recall task* (Colle and Welsh 1976; Salamé and Baddeley 1982), each trial started with a 1000 ms preparation interval (showing an animated blue square decreasing in size) followed by a random sequence of eight randomly drawn digits (from 1–9). Each digit was presented for 1000 ms, immediately followed by the next digit, and there was an additional 6000 ms retention interval showing a blank screen. To measure the degree of interference by task-irrelevant sound with serial recall, either a passage of free-running Finnish speech (which the participants did not understand) spoken by a male voice (a weather forecast; taken from Kattner and Ellermeier 2014) or white noise was presented during both the presentation and the retention interval. After the retention interval, participants were asked to recall the digits in correct serial order by clicking on the numbers in a 3 × 3 numeric pad shown on the screen. Feedback indicating the number of correctly recalled digits was presented for 1000 ms before the next trial started (feedback was presented in green font when 5 or more digits were recalled, and in red when less than 5 digits were recalled). The entire task consisted of 12 trials with irrelevant speech and 12 trials with irrelevant noise which were presented in randomized order.

For the 200 *task-switching* trials*,* a random letter (A, E, I, O, K, L, M, or P) and a random number (2–9) were presented on the screen, and participants were asked to categorize either the letter or the number in a typical alternating-runs procedure (Kattner et al. 2019; Rogers and Monsell 1995) providing an equal number of switch (second and fourth trial) and repeat trials (first and third trial). Specifically, participants indicated whether the letter was a vowel or consonant, and whether the number was even or odd by pressing the left or right arrow key on the keyboard. Participants were instructed to respond as fast as possible. Both the accuracy and the actual response time were shown as feedback for 750 ms after each response (e.g. “Falsch! 1689 ms” [Incorrect! 1689 ms]). No feedback but a prompt to be faster was provided if no response was given within 5000 ms.

For both the pre- and the post-test sessions, eighteen unique problems of increasing difficulty were selected from the 36-item short form of *Ravens’s Advanced Progressive Matrices* (odd item numbers at pre-test and even item numbers at post-test). Each matrix problem was presented on the screen and participants were asked to choose the stimulus which completes the matrix based on a to-be-identified rule. A set of eight response options was shown below the matrix (together with numbers), and responses were made by pressing the respective number on the keyboard. Knowing that the difficulty of problems increases, participants were given ten minutes to complete the eighteen problems one after another without the option to return to a previous problem. The remaining time (in seconds) was shown at the top of the screen. No feedback was provided during or after the task.

### Training tasks

The *n*-back group was trained with an adaptive version of the dual *n*-back task (Jaeggi et al. 2007, 2008; Salminen et al. 2016) for eight 90-min sessions (including breaks and instructions). In this task, a running sequence of visual squares and auditory letters was presented. On each trial, a bluish square (1° diameter) was presented at one of eight possible locations on the screen (at 4° eccentricities from the fixation) while a spoken consonant (B, F, J, K, L, T, or V; male voice) was presented simultaneously via headphones. A new square and a new consonant was presented every second, and participants were asked to hit the space bar on the keyboard whenever one of the present two stimuli (square or letter) matched with the stimuli that were presented *n* trials back in the sequence (*n* was the same for squares and letters). No response was required if both the square and the letter were different from the stimuli *n* trials back. Both the square and the letter were drawn randomly to be identical to the ones *n* trials back with independent probabilities of *p* = 0.15 each, so participants should be required to make a response in about 27.7% of the trials, and not to respond on about 72.3% of the trials (based on simulations with the *rand* function conducted in Matlab). Responses had to be given rapidly within the 1000 ms of stimulus presentation. Text feedback was provided in case of incorrect (‘Finger weg!’ [Hands off]) or missed responses (‘Verpasst!’ [Missed]). Each training session started with *n* = 1, but task difficulty was changed with the participants’ performance. Specifically, the value of *n* was increased by 1 if participants were at least 90% correct in a block of 25 + *n* trials. The value of *n* was decreased by 1 if accuracy was at or below 70%, and *n* did not change if accuracy was between 70 and 90%. Participants could take a short break every 20 min. Each training session continued until either 80 min had passed (break times not included) or 1500 trials were completed (only 13.0% of participants in the *n*-back training group and no participant in the inhibitory *n*-back group completed 1500 trials within 80 min).

The inhibitory *n*-back group was trained on a similar task as the *n*-back group, with the same random sequences of squares and letters being presented. In contrast to the standard *n*-back group, participants of the inhibitory *n*-back group were asked to hit the space key as fast as possible on every trial except when either the location of the square or the spoken letter was identical to the ones *n* trials back in the sequence. As the independent probabilities of the square and the letter to be identical to the ones *n* trials back were the same as in the *n*-back training group (i.e., *p* = 0.15), participants were supposed to respond in about two thirds of the trials, and not to respond in about one third of the trials. Thus, in the inhibitory dual *n*-back task, pressing the space bar is supposed to be the pre-potent response, which is required on the majority of trials, and participants are required to suppress this response only on the less frequent *n*-back trials. With equal demands for working memory updating (and other control processes possibly involved), this task is assumed to involve a greater demand for inhibitory control than the standard *n*-back task.

## Results

The individual data from the two *n*-back trainings and the various measures obtained with the tasks at pre-test and post-test are openly available as csv files in an OSF repository: https://osf.io/ubxap/?view_only=d200d4917bc945c585913b069631e55a.

### Performance during training

Across all participants 24.6% (*SD* = 1.4%) of the trials in the *n*-back group and 24.7% (*SD* = 1.1%) of the trials in the inhibitory *n*-back group were “*n*-back trials” in which either the auditory or the visual item (or both) was identical to the one n trials back. Accordingly, participants in the *n*-back group responded with a key press on 23.3% of the trials (*SD* = 6.3%), whereas participants in the inhibitory *n*-back group responded with a key press on 82.1% of the trials (*SD* = 6.4%). Hence, in contrast to the *n*-back group, a key press was the dominant response in the inhibitory *n*-back group, suggesting that inhibitory control is required to suppress the key press on the “*n*-back trials”. Across all eight training sessions, participants of the *n*-back group completed *M* = 10,960 *n*-back trials (*SD* = 364), whereas participants of inhibitory *n*-back group completed *M* = 10,306 trials (*SD* = 1719), but this difference was not statistically significant, *F*(1,46) = 3.20; *p* = 0.08; *η*^2^_G_ = 0.06.

**Fig. 1:**
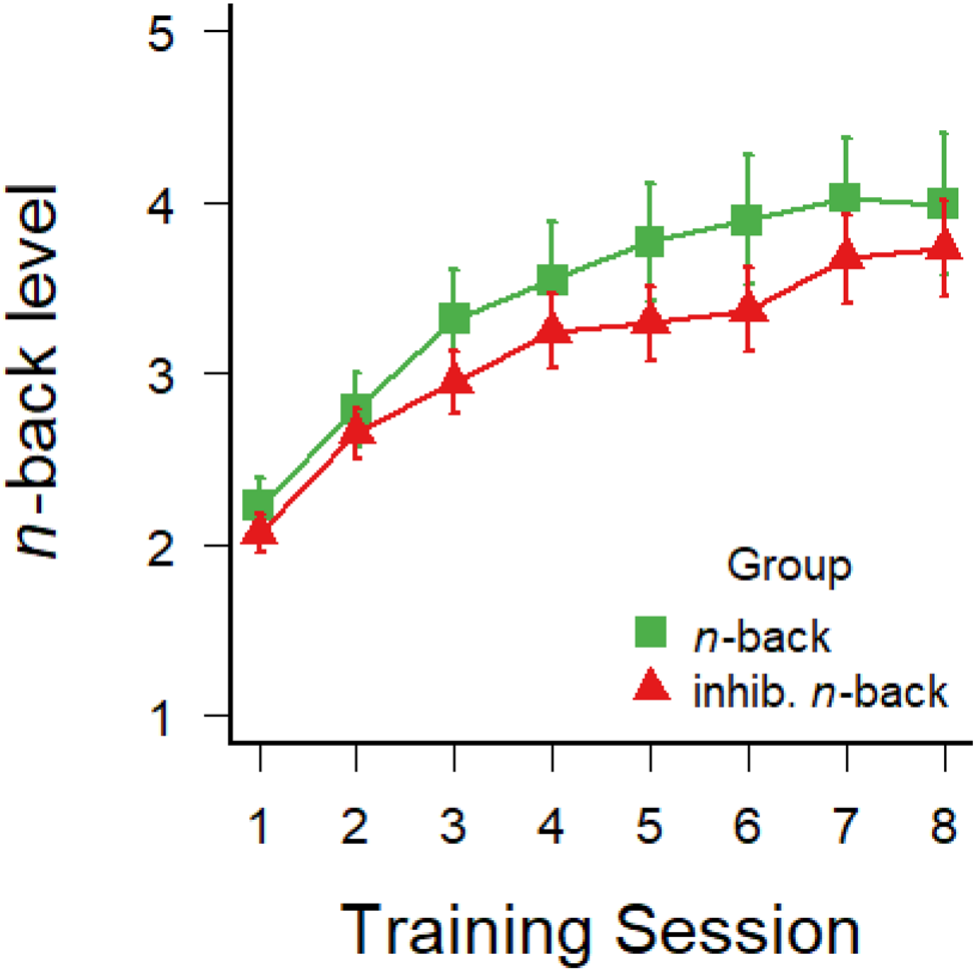
Average number n of to-be-remembered items across the eight sessions of dual *n*-back training in the *n*-back and inhibitory *n*-back groups, keeping the accuracy of responses between 70 and 90%. Error bars depict ± 1 standard errors of the mean

Figure 1 illustrates that the average level of *n* increased across the eight training sessions both in the *n*-back and inhibitory *n*-back groups, demonstrating that the participants’ *n*-back working-memory updating span increased with both types of training. A 2 (group) × 8 (training session) mixed-factors ANOVA with training session as a repeated-measures factor confirmed this improvement with a significant main effect of training session, *F*(7,315) = 46.01; *p* < 0.001; *η*^2^_G_ = 0.16. There was no significant main effect of group, *F*(1,45) = 0.71; *p* = 0.40; *η*^2^_G_ = 0.01, and no group × session interaction, *F*(7,315) = 0.49; *p* = 0.84; *η*^2^_G_ < 0.01, suggesting that both average performance and the rate of learning did not differ between the two training groups.

### Transfer to working memory updating

**Fig. 2:**
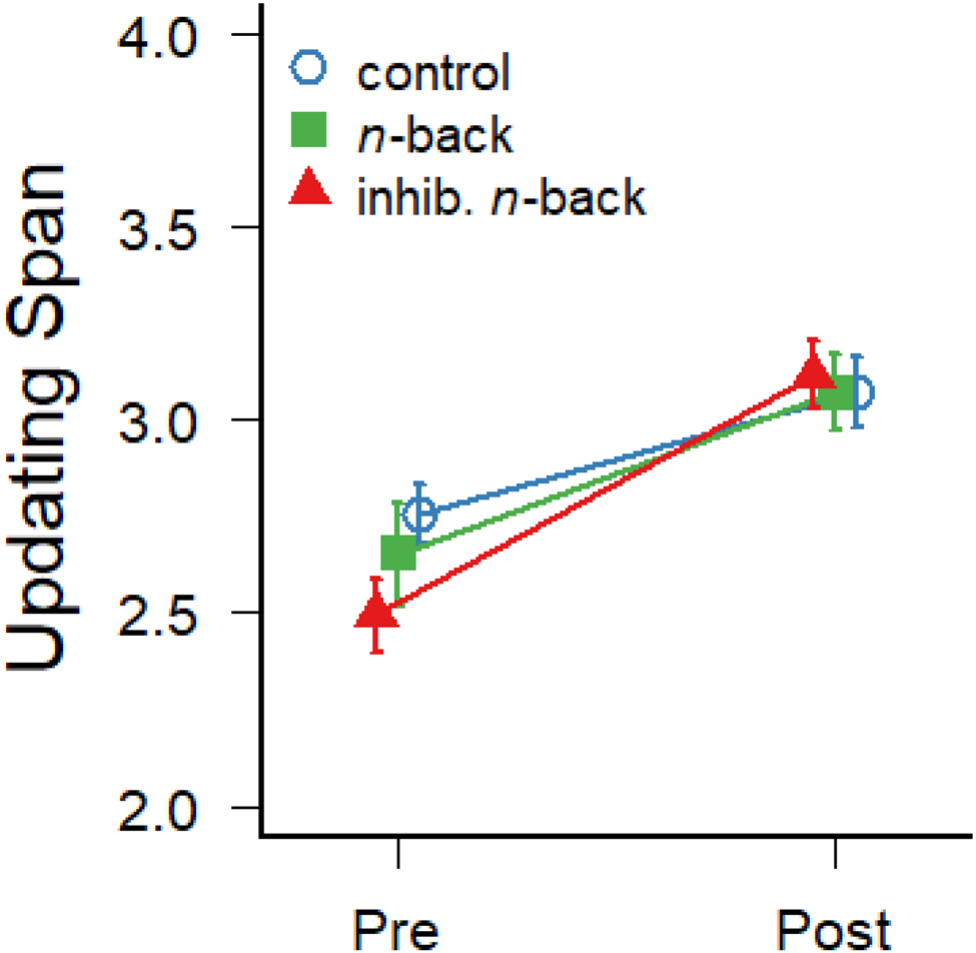
Average number of correctly recalled items in the four-color updating task at pre- and post-test. Error bars depict ± one standard error of the mean

Transfer effects of the two types of trainings were first assessed with regard to the working memory span in an untrained color updating task. The average number of correctly recalled colors in the updating task is illustrated in Fig. 2 for the three experimental groups. Intra-class correlations between the average working memory updating span at pre- and post-test (calculated with the {irr} package in R) demonstrated good test–retest reliability, ICC(C,2) = 0.73 (*N* = 74). Interestingly, all groups significantly improved their color updating span from pre-test (*M* = 2.64; *SD* = 0.51) to post-test (*M* = 3.09; *SD* = 0.46), *F*(1,71) = 79.35; *p* < 0.001; *η*^2^_G_ = 0.18. However, the improvement also differed between groups, as suggested by the significant interaction, *F*(2,71) = 3.25; *p* = 0.04; *η*^2^_G_ = 0.02. Pairwise *t *tests on the increments of working memory updating spans, corrected for multiple comparisons (Benjamini and Hochberg 1995), revealed a significant difference between the inhibitory *n*-back group and control group, *p* = 0.04, but no difference between the inhibitory *n*-back and standard *n*-back groups, *p* = 0.17, and the standard *n*-back and control groups, *p* = 0.41. In addition, Bayes factors (calculated with the R package {BayesFactor}; Rouder et al. 2009) indicate that it is about three times more likely that the inhibitory *n*-back produced a greater increment of updating span than in the control group (BF_10_ = 3.08), whereas the effect of the standard *n*-back training does not seem to differ from the control group (BF_10_ = 0.37). This suggests that the enhanced demands for inhibitory control during training in the inhibitory *n*-back group produced more transfer to general updating abilities than the training with the standard dual *n*-back task. There was no main effect of group on the updating span, *F*(2,71) = 0.41; *p* = 0.66.

### Transfer to response inhibition

**Fig. 3:**
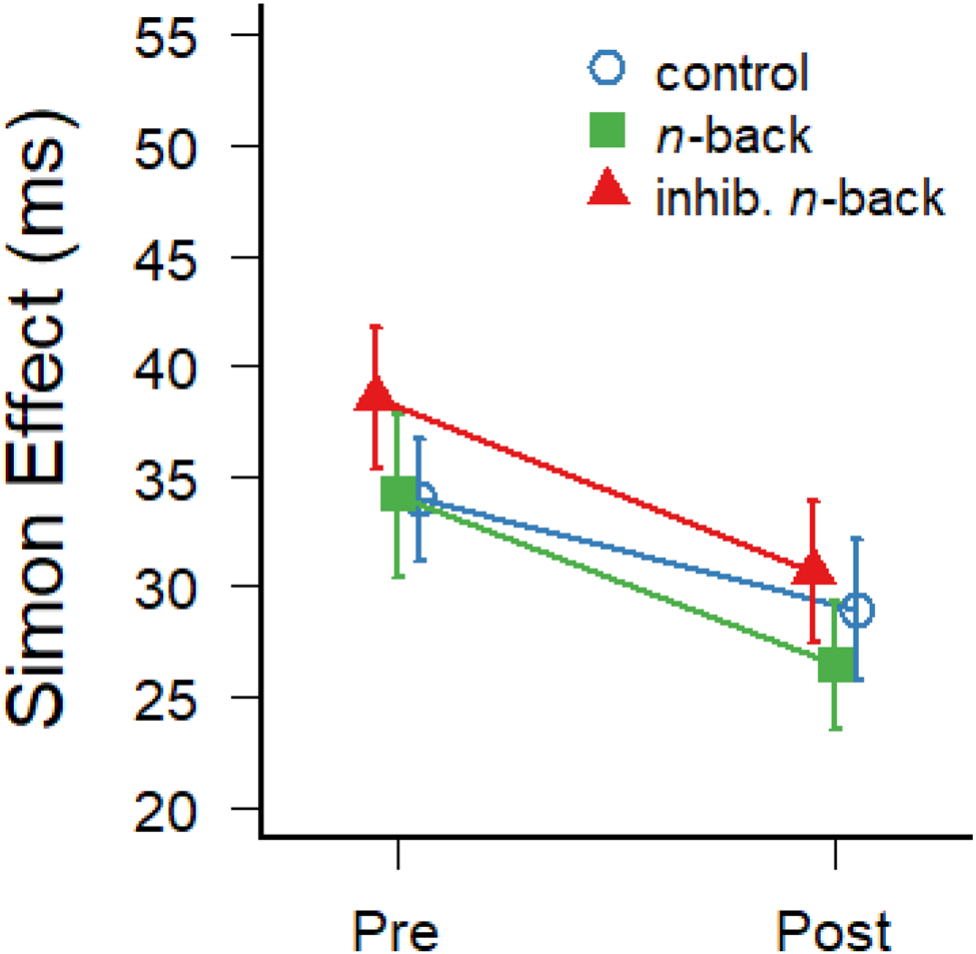
Average response time differences between spatially compatible and incompatible trials in the Simon task at pre- and post-test. Error bars depict ± one standard error of the mean

Transfer was further assessed with regard to response inhibition using the Simon task which requires inhibition of spatially compatible (pre-potent) responses. The strength of response inhibition was measured by subtracting the mean response times on spatially compatible trials from spatially incompatible trials (on which the pre-potent spatial response needs to be inhibited). Trials with incorrect responses or with outlier response times longer than 1.5 interquartile ranges above the 75% percentile of the entire RT distribution (> 572 ms, 4.6% of all trials) were removed prior to the analysis. The resulting Simon effects at pre- and post-test are illustrated separately for the three experimental groups in Fig. 3. The average Simon effects at pre- and post-test demonstrated moderate test–retest reliability, ICC(C,2) = 0.68 (*N* = 74).

A 3 (group) × 2 (test: pre, post) mixed-factors ANOVA on Simon effects revealed a main effect of test, *F*(1,71) = 14.11; *p* < 0.001; *η*^2^_G_ = 0.05, with an overall decrease of the Simon effect from pre-test (*M* = 35.5 ms; *SD* = 15.9 ms) to post-test (*M* = 28.7 ms; *SD* = 15.4 ms), most likely demonstrating a general practice effect for response inhibition. However, there was no main effect of group, *F*(2,71) = 0.65; *p* = 0.53; *η*^2^_G_ = 0.01, and no interaction between group and test, *F*(2,71) = 0.27; *p* = 0.76; *η*^2^_G_ < 0.01, suggesting that the present two trainings with dual *n*-back tasks did not reduce the stimulus-response incompatibility effect, as compared to a passive control group. This interpretation is confirmed also by the Bayes factors indicating that it is more likely that the decrease of the Simon effect did not differ between the two training groups and the control group (BF_10_ = 0.34 and BF_10_ = 0.33 for the inhibitory and standard *n*-back training groups, respectively). Hence, in particular the inhibitory *n*-back training does not seem to have strengthened the inhibition of pre-potent responses in general.

### Transfer to auditory distraction

**Fig. 4:**
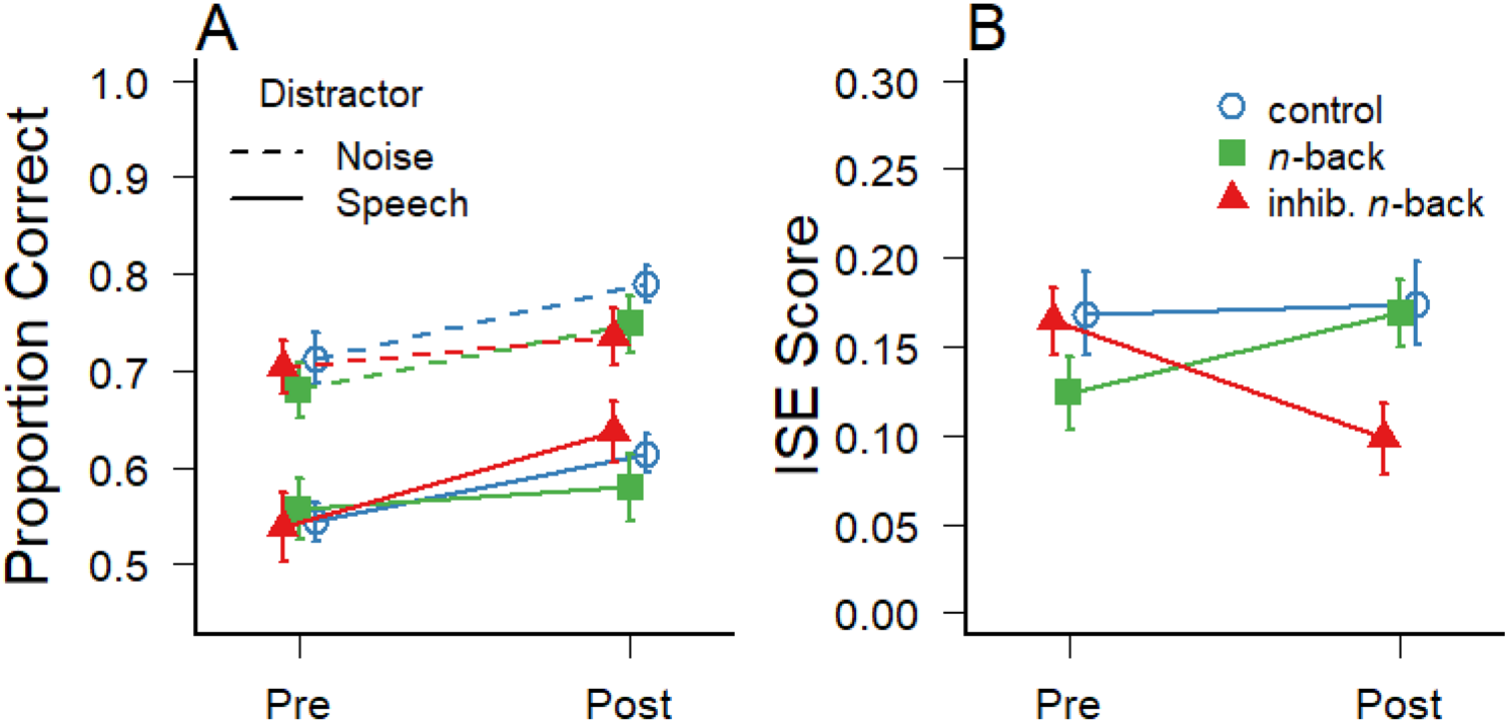
**a** Average serial recall performance (proportion of digits recalled in the correct serial position) on trials with task-irrelevant speech and noise at pre- and post-test, and **b** the difference scores in recall performance between speech and noise trials referring to the degree of auditory distraction (ISE score). Error bars depict ± 1 standard errors of the mean

The average proportion of recalled items on trials with task-irrelevant speech and noise before and after training is illustrated in Fig. 4a. As a measure of the irrelevant speech effect (ISE score), difference scores were calculated by subtracting performance during speech from performance during noise. These ISE scores demonstrated moderate test–retest reliability for the entire sample, ICC(C,2) = 0.46 (*N* = 74). However, the disruptive effect of irrelevant speech appears to have decreased from pre-test to post-test in the inhibitory *n*-back group, but not in the standard *n*-back training group and the control group (see the ISE scores in Fig. 4b). This observation was confirmed by a significant three-way interaction between sound, group and test on verbal recall accuracy, *F*(2,71) = 5.44; *p* = 0.01; *η*^2^_G_ = 0.01, suggesting that the cognitive training with enhanced demands for inhibition enhanced the participants’ resistance to distractor interference in the verbal short-term memory task. Pairwise *t* tests of the decrements of ISE scores (corrected for multiple comparisons) confirmed a significant contrast between the inhibitory *n*-back training group and both the standard *n*-back training group (*p* = 0.01) and the control group (*p* = 0.05), whereas there was no difference between the standard *n*-back training and the control group (*p* = 0.25). Likewise, Bayes factors indicate that a differential decrease of the ISE from pre-test to post-test in the inhibitory *n*-back training group, compared to the two other groups, is much more likely than the null hypothesis (BF_10_ = 12.56), whereas there is no evidence for a difference in the change of ISE scores between the standard *n*-back training and the control group (BF_10_ = 0.50).

There was also a significant main effect of sound, *F*(1,71) = 229.30; *p* < 0.001; *η*^2^_G_ = 0.24, confirming the overall irrelevant speech effect. In addition, there was a main effect of test, *F*(1,71) = 28.04; *p* < 0.001; *η*^2^_G_ = 0.05, indicating a general improvement in the serial recall task (from *M* = 0.62; *SD* = 0.13 at pre-test to *M* = 0.68; *SD* = 0.12 at post-test). The ANOVA revealed no other significant effects, *F* < 1.45; *p* > 0.24.

### Far transfer to task-switching and fluid intelligence

Transfer of working memory training was assessed also with regard to tasks that require cognitive set shifting and general problem-solving abilities.

**Fig. 5:**
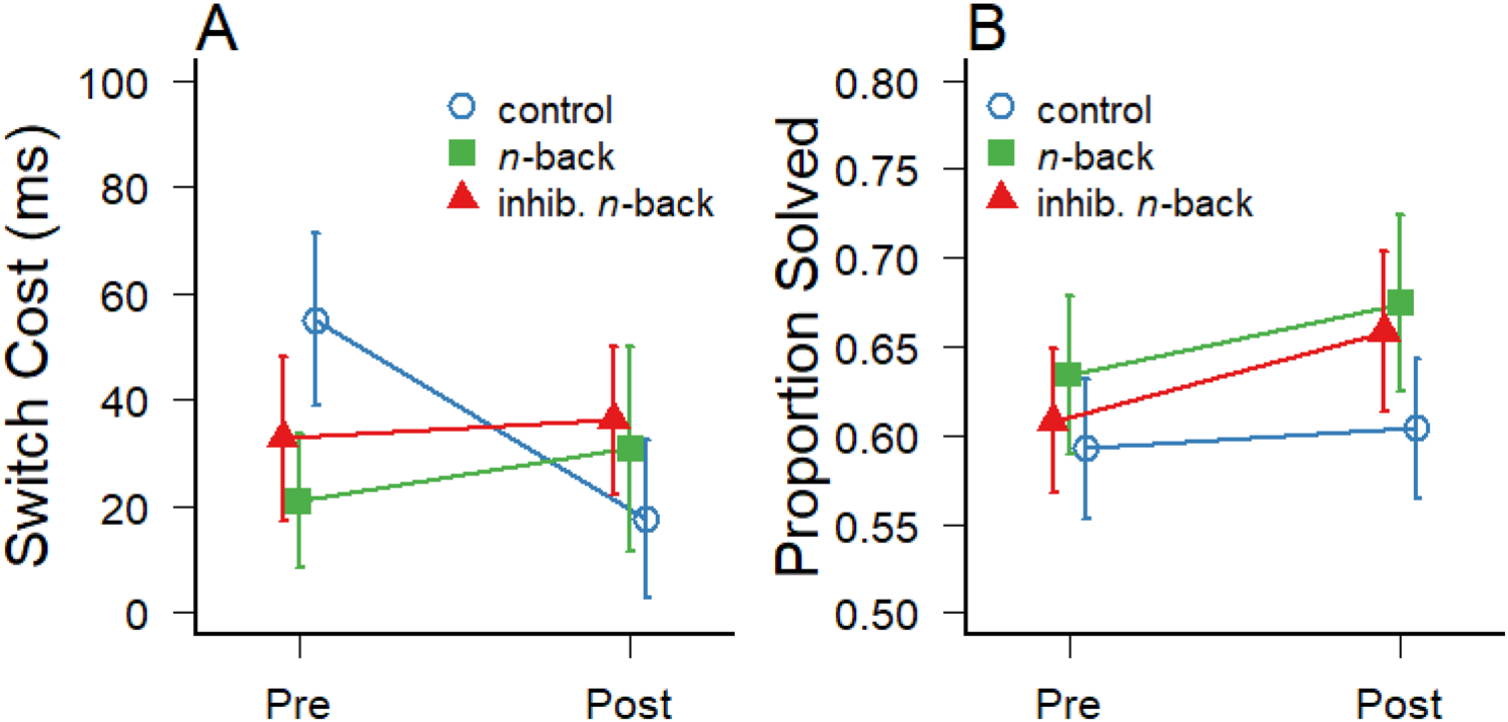
**a** Response-time costs of task switches (i.e., response times on repeat trials subtracted from response times on switch trials), and **b** proportion of solved matrix problems in Raven’s Advanced Progressive Matrices test of fluid intelligence at pre- and post-test. Error bars represent ± 1 standard error of the mean

For the task-switching data, response times during task-switching were analyzed only for trials with correct responses. In addition, outlier response times longer than 1.5 interquartile ranges above the 75% percentile of the entire RT distribution (> 2230 ms, 5.6% of all trials) were removed prior to the analysis. The response-time costs due to switching from one task set (letter categorization) to a different task set (number categorization) were calculated by subtracting the response times on repeat trials from switch trials. The resulting switch costs are illustrated in Fig. 5a, and the average switch costs demonstrated good test–retest reliability between pre- and post-tests, ICC(C,2) = 0.68 (*N* = 74). A 2 (test) × 3 (group) mixed-factors ANOVA revealed no main effect of group, *F*(2,71) = 0.16; *p* = 0.85; *η*^2^_G_ < 0.01, no main effect of test, *F*(1,71) = 1.10; *p* = 0.30; *η*^2^_G_ < 0.01, but a marginally significant group × test interaction, *F*(2,71) = 3.07; *p* = 0.05; *η*^2^_G_ = 0.02. The interaction is likely driven by the reduction of the relatively high switch in the control group, whereas the two working memory trainings did not reduce the participants’ costs during task-switching (see Fig. 5a), suggesting that the two types of *n*-back training did not affect set shifting abilities. Moreover, pairwise t tests and Bayes factors revealed no clear evidence for a differential change in switch costs between the control group and the two *n*-back training groups (adjusted *p* = 0.08; BF_10_ = 1.39 and BF_10_ = 1.62, respectively).

Finally, transfer was assessed with regard to non-verbal problem-solving capabilities related to fluid intelligence by having participants solve eighteen different visual matrix problems at pre- and post-test. The average number of problems solved within 10 min is shown in Fig. 5b, and these scores of fluid intelligence demonstrated good test–retest reliability across all participants, ICC(C,2) = 0.83 (*N* = 74). While there seems to be a slight increase of the scores in the two trained groups, compared to the control group, a 2 (test) × 3 (group) mixed-factors ANOVA revealed no main effect of group, *F*(2,71) = 0.51; *p* = 0.61; *η*^2^_G_ = 0.01, and no significant interaction, *F*(2,71) = 0.38; *p* = 0.68; *η*^2^_G_ < 0.01, suggesting that the two types of working memory training did not exert a reliable effect on fluid intelligence in the present study. There was only a marginally significant main effect of test, *F*(1,71) = 3.13; *p* = 0.08; *η*^2^_G_ = 0.01, indicating a trend for a general pre-post improvement in matrix solving performance across all three groups. Consistent with these results, corrected pairwise *t* tests and Bayes factors suggest that it is unlikely that the type of training differentially affected the change of fluid intelligence scores from pre-test to post-test (*p* > 0.80 and BF_10_ < 0.37 for the three contrasts).

## Discussion

The present study showed that an extended cognitive training with two adaptive versions of the demanding dual *n*-back task, varying in the degree of inhibitory control required, improved working memory capacity not only for the trained task (i.e., the value of *n* increased from the first to the eighth training session), but also for a different type of updating task. However, this improvement on the untrained memory updating task was significantly different from the passive control group (which showed general improvement on the task) only for participants who were trained on the dual *n*-back task with additional demands for response inhibition, but not for participants who were trained on the standard dual *n*-back task. Hence, the transfer of a training on the dual *n*-back task to general working memory updating abilities seems to depend on the degree of inhibitory control involved in the training task. This finding is quite consistent with a recent meta-analysis on the effects of *n*-back training concluding that the magnitude of transfer from training on the standard dual *n*-back training to other working memory paradigms, such as operation span or running span tasks, is very small (Soveri et al. 2017). The present findings suggest that an extensive training with cognitive tasks for which multiple executive functions are required (e.g., updating and inhibition) may enhance the likelihood of near transfer effects to other working memory tasks, as compared to a cognitive training with tasks which address only a single executive function (e.g., working memory updating in case of the *n*-back task).

In addition to the near transfer effects of the present training to working memory updating, transfer was tested also with regard to two separate forms of inhibition. As the two types of *n*-back training tasks differed only with regard to the demand for inhibition control (i.e., to suppress the predominant response on *n*-back trials), more transfer to other inhibition tasks was expected for participants who were trained on the inhibitory dual *n*-back task, as compared to a training on the standard dual *n*-back task. However, it was unclear whether to expect transfer to inhibitory control of pre-potent responses, resistance to distractor interference, or both (Friedman and Miyake 2004). The results indicate that the two training tasks did not differ with regard to transfer to pre-potent response inhibition. In fact, none of the two *n*-back trainings reduced the response-time costs for key presses that were spatially incompatible with the target location in the Simon task, as compared to the passive control group. This finding may be surprising given that the inhibitory dual *n*-back task required participants to suppress the more frequent and hence pre-potent key presses throughout the eight training sessions. However, the training task did not require participants to solve a spatial compatibility conflict as in the Simon task. Hence, the present results suggest that the inhibition of a frequently occurring response (as during training) may depend on a form of inhibitory control that is functionally distinct from the inhibition that is required for the resolution of a stimulus-response compatibility conflict. Future research is required to determine whether the present training of inhibition within a dual *n*-back working memory task generalizes to more similar types of pre-potent response inhibition tasks, such as the stop-signal task (Logan 1994; Verbruggen and Logan 2009).

By contrast, the two types of *n*-back training yielded differential transfer effects with regard to inhibitory control of the interference produced by task-irrelevant speech distractors in a verbal short-term memory task. Specifically, participants who were trained with the newly developed inhibitory dual *n*-back task, requiring continuous updating and inhibition of the contents in working memory, seem to have enhanced their resistance to irrelevant speech during serial recall. In contrast, a working-memory training with the standard dual *n*-back task demanding less inhibitory control does not seem to have an effect on the magnitude of the irrelevant speech effect. This attenuation of auditory distraction after inhibitory *n*-back training suggests that the training-related strengthening of inhibition enabled participants to reduce the interference from the external auditory environment (Friedman and Miyake 2004). With regard to accounts of auditory distraction, the finding indicates that inhibitory control may have prevented attentional capture by task-irrelevant speech, but it may not have reduced the disruptions due to interference-by-process (e.g., in line with the duplex-mechanism account; Hughes 2014). Specifically, the fact that the irrelevant speech effect was reduced, but not eliminated after a comprehensive inhibitory-control training indicates that enhanced inhibitory control may prevent the diversion of attention by irrelevant speech, whereas the (remaining) interference with the seriation process produced by the changing-state nature of irrelevant speech may not be susceptible to top-down control. The observation that a considerable portion of auditory distraction (presumably the changing-state effect) could not be eliminated by enhanced inhibitory control might suggest that the interference-by-process mechanism is not related to general working memory functions, and thus not susceptible to inhibitory or cognitive control (see Hughes 2014). This interpretation of the present results would be consistent also with other recent observations showing that only attentional capture, but not the changing-state effect, can be reduced through cognitive control (Hughes et al. 2013; Hughes and Marsh 2020a; Marsh et al. 2019), and is related to working memory capacity (Beaman 2004; Sörqvist 2010). The findings also fit very well with recent observations of a the irrelevant speech effect to be reduced (but not eliminated) after a training of auditory selective attention (using a dichotic-listening task; Kattner and Ellermeier 2020), indicating that the portion of the irrelevant speech effect which can be attributed to attentional capture may be susceptible to attentional control.

Nevertheless, since the irrelevant speech effect may comprise both attentional capture and interference-by-process mechanisms of auditory distraction, it is still possible that inhibitory control also resolves the (presumably uncontrollable) interference between changing-state sound and serial-order processing. Of course, the fact that the irrelevant speech effect was only attenuated, but not eliminated, after eight sessions of inhibitory-control training does not prove that it is the changing-state effect, which remained. Moreover, it could be argued that eight sessions of inhibitory control training may not be sufficient to eliminate the disruptive effect of irrelevant changing-state sound (an effect which appears to be very robust having survived years of every-day mental activities requiring the inhibition of irrelevant information). For instance, there is evidence that irrelevant speech does not interfere at all with short-term memory in congenitally and early blind individuals (Kattner and Ellermeier 2014), suggesting that enhanced inhibitory control of auditory information resulting from a life-long experience with a primarily auditory environment may eliminate the interference-by-process portion of auditory distraction as well. Further research is required to determine whether the attenuation of the irrelevant speech effect in the present study was due to an attenuation of attentional capture or the task-specific interference-by-process. This could be accomplished, for instance, by contrasting the transfer effects of an inhibitory control training on the disruption produced by auditory deviants (which should be due to attentional capture alone) and changing-state sound (which should reflect interference-by-process). Alternatively, transfer effects could be investigated with regard to auditory distraction in non-serial short-term memory tasks, which are known to be immune to a changing-state effect (e.g., the missing-item task; Beaman and Jones 1997). If attentional capture depended on the strength of inhibitory control, then the often relatively small disruptive effect of irrelevant speech in the missing-item task (due to a diversion of attention) might be eliminated completely as a result of inhibitory *n*-back training (compare Hughes and Marsh 2020b for a similar observation with regard to the effect of foreknowledge).

More generally, the present results indicate that an extensive cognitive training cannot be used only to enhance working memory updating (Dahlin et al. 2008a, b) and set shifting (Pereg et al. 2013), but also to strengthen the inhibitory-control function of working memory in terms of the inhibition of auditory distractor interference. The present study is the first to demonstrate that the extent of auditory distraction in short-term memory can be reduced experimentally through a working memory training with enhanced demands for inhibitory control in two stimulus modalities (i.e., inhibition of responses to visuospatial and auditory stimuli in the dual *n*-back task). In contrast, the same working memory training with reduced demands for inhibitory control did not affect auditory distraction. Hence, the pattern of results indicates that the training-related decrease of interference by task-irrelevant auditory stimuli was not driven by working memory capacity in general, but rather by a specific inhibitory-control function (i.e., resistance to distractor interference; Friedman and Miyake 2004).

Finally, the present study also investigated possible far transfer effects of an extended dual *n*-back training on (a) set shifting abilities and (b) fluid intelligence scores. Regardless of the degree of inhibitory control involved, the present dual *n*-back training did not reduce the response-time costs resulting from switching between two different categorization tasks. This suggests that training on the *n*-back task does not enhance executive set shifting abilities. Moreover, in contrast to previous findings (Jaeggi et al. 2008), the dual *n*-back training did not affect fluid intelligence in the present study. Specifically, participants in all experimental groups were able to solve about 9.5–10 out of 18 problems of the short version of Raven’s Advanced Progressive Matrices test at pre-test (which is equivalent to the pre-test scores reported by Jaeggi et al. 2008). In contrast to the control group and the inhibitory dual *n*-back training group, the average fluid intelligence score was slightly enhanced at post-test for participants who were trained on the standard dual *n*-back task, but the group differences in gains on fluid intelligence did not turn out to be statistically significant. In line with several other recent findings of a lack of “far transfer” (Harrison et al. 2013; Melby-Lervåg et al. 2016; Redick et al. 2013), the present result seems to contradict the findings reported by Jaeggi et al. (2008). However, the absence of a transfer effect to fluid intelligence might also be due to differences in the spacing of training times. Specifically, participants in the present study were trained for eight 80-min training sessions (breaks not included), whereas Jaeggi et al. (2008) trained participants for either eight, twelve, seventeen or nineteen 25-min sessions. In the study by Jaeggi et al. (2008), the training-related gain on fluid intelligence was shown to increase with the number of training sessions, and statistically significant gains of fluid intelligence relative to pre-test were found only after seventeen and nineteen 25-min sessions of training, but not after eight and twelve sessions of training. Hence, it seems that the transfer to fluid intelligence depends on the training dosage. However, in the present study, participants were trained for 640 min in total (8 × 80 min), so the total training time exceeded the nineteen training sessions in the Jaeggi et al. (2008) study (i.e., 475 min). The fact that no reliable transfer to fluid intelligence was observed in the present study suggests that temporal spacing of training sessions (i.e., multiple short sessions) may enhance the chances of far transfer effects, as compared to massed training sessions (i.e., few long sessions).

Taken together, the present study shows that working memory capacity can be enhanced successfully with an extended training on two different types of dual *n*-back tasks varying to the degree of inhibitory control required. In general, transfer to cognitive abilities that are not directly related to the training task was very limited. However, in contrast to the standard *n*-back task with relatively low demands for inhibitory control, training on the newly developed inhibitory dual *n*-back task was found to reduce the degree of interference produced by irrelevant speech in a serial short-term memory task. This finding indicates that the inhibitory dual *n*-back task enhanced not only working memory updating abilities, but also inhibitory control of distractor interference, thus enabling more transfer to tasks for which these executive functions are required (e.g., inhibited processing of task-irrelevant speech). More research is required to disentangle the effects of enhanced inhibitory control on attentional capture and interference-by-process mechanisms of auditory distraction, and to assess possible transfer effects of an inhibitory working memory training on other forms of inhibitory control, such as pre-potent response inhibition or inhibition of proactive interference in memory.
